# Biotechnological Potential of Lactic Acid Bacteria Isolated From Ethiopian Honey Wine, *Tej*

**DOI:** 10.1155/ijm/4014096

**Published:** 2025-08-11

**Authors:** Minyilal Sisay, Asnake Desalegn, Fitsum Tigu, Mogessie Ashenafi, Feng-Yan Bai, Dagim Jirata Birri

**Affiliations:** ^1^Department of Microbial Sciences and Genetics, College of Natural and Computational Sciences, Addis Ababa University, Addis Ababa, Ethiopia; ^2^Bio and Emerging Technology Institute, Addis Ababa, Ethiopia; ^3^State Key Laboratory of Mycology, Institute of Microbiology, Chinese Academy of Sciences, Beijing, China; ^4^Center for Food Security Studies, College of Development Studies, Addis Ababa University, Addis Ababa, Ethiopia

**Keywords:** acid–bile tolerance, antimicrobial, antibiotic susceptibility, anticholesterol, antioxidant, lactic acid bacteria (LAB) probiotics and Ethiopian honey wine, *Tej*

## Abstract

Some members of the lactic acid bacteria (LAB) have been used as probiotics. Ethiopian honey wine, *Tej*, may be a useful source of potential probiotic bacteria. However, LABs from this source have not yet been evaluated for their probiotic properties. This study was conducted to evaluate the *in vitro* probiotic properties of LAB isolated from Ethiopian honey wine, *Tej*. For this purpose, 30 samples were collected from Southwest Ethiopia. LAB isolates were first tested for their antimicrobial activity and those with this property were evaluated for probiotic properties, such as tolerance to acid, salt, and bile, adherence properties, anticholesterol activity, antioxidant activity, and antibiotic susceptibility. The antibacterial activity of the LAB isolates against test organisms was assessed by the agar well diffusion method. Acid, salt, and bile tolerance were evaluated by the plate count method. Adhesion properties were assessed by the determination of bacterial hydrophobicity to the nonpolar solvent p-xylene. Anticholesterol was determined by measuring the remaining cholesterol in the spent broth. The antioxidant capacity was assessed by the 1,1-diphenyl-2-picrylhydrazyl free radical-scavenging capacity, and the antibiotic susceptibility of isolates was tested by the disk diffusion method. A total of 143 LABs were isolated from *Tej* samples. The LAB count was ranged between 7.4 and 6.5 log cfu/mL. Of the 143 LAB isolates, 37 exhibited different levels of antimicrobial activity. Eight of these were identified to species level by 16S ribosomal genes sequence analysis. The greatest inhibition was against *Shigella boydii* ATCC 25931 with 19 ± 4.2 mm, and the least inhibitory activity was against *Escherichia coli* ATTC 25922 and *Staphylococcus aureus* ATTC 25913 with 8.0 ± 1.4. All except two isolates survived at pH 2 and pH 3 (18.4%–89.6%). Then, 37 isolates survived in more than 50% bile and 54%–67% adhesion capacity. The cholesterol-lowering and 1,1-diphenyl-2-picrylhydrazyl free radical-scavenging capacities ranged from 8% to 54% and 13% to 33%, respectively. Most isolates were susceptible to antibiotics, except for one isolate that resisted all tested antibiotics. This study shows that many LABs isolated from Ethiopian honey wine, *Tej* have probiotic properties and they can be considered as probiotic candidates. We recommend evaluation of *in vivo* probiotic properties of the LAB isolates to provide strong supporting evidence.

## 1. Introduction

Lactic acid bacteria (LABs) are Gram-positive, nonspore forming rod or cocci, catalase-negative bacteria that produce lactic acid as a main product of hexose catabolism. LABs are found in different fermented foods and drinks such as beer, milk, bread, cassava. and pickled (fermented) vegetables [1]. LABs include *Enterococcus*, *Lactobacillus*, *Streptococcus*, *Lactococcus*, *Pediococcus*, and *Leuconostoc* and many other genera [2]. Lactic acid bacterial strains isolated from different food sources are generally recognized as safe (GRAS). They are commonly used as probiotics to facilitate certain biological functions of the host [3]. LAB have various potential health benefits and functional roles in human or animal health including immunomodulatory properties, antimicrobial, anticancer, antioxidant activity, antiulcer, and cholesterol-lowering properties [4–6]. Safe transit through the stomach, and survival and colonization in the intestinal tract are critical and foremost parameters to qualify as a potential candidate for further screening for probiotic attributes [7]. Tolerance towards bile acids is attributed to presence of bile salt hydrolases (BSHs), product of *bsh* gene of bacteria [8]. According to Girum et al. [9], LABs such as *Lactobacillus*, *Pediococcus*, *Streptococcus*, and *Leuconostoc* species isolated from traditional Ethiopian fermented beverages show good antagonistic activity against various food-borne pathogens [10]. Such antagonistic property is attributed to their ability to lower pH (produce acid) and inhibiting a variety of metabolic functions. A number of studies showed the probiotic properties of LAB isolated from various Ethiopian traditional fermented foods and alcoholic beverages such as data, awaze, shamita, and kocho [11, 12]. However, there are a few research findings on the probiotic activity of LAB isolated from Ethiopian honey wine, *Tej*, produced in Southwest, Ethiopia. Therefore, the main aim of this study is to evaluate LAB isolated from Ethiopian honey wine, *Tej*, for probiotic properties.

## 2. Methods

### 2.1. The Study Area

The study was conducted in four areas in south west Ethiopia: Jimma, Kafa, Bench Maji, and Sheka zones. Jima zone is located in Oromia regional state, whereas the latter three zones are situated in South West Ethiopia Regional state.

### 2.2. Sample Collection

A total of 30 fermented Ethiopian honey wine, *Tej*, samples were collected in sterile bottles from different well-known hotels and honey wine bars found in the study areas. The samples were transported to Addis Ababa University Microbiology laboratory and stored in the refrigerator at 4°C until analysis was carried out.

### 2.3. Determination of pH, Moisture Content, and Titratable Acidity of Ethiopian Honey Wine, *Tej*

The pH of Ethiopian honey wine, *Tej*, samples was measured by digital pH meter. Moisture content was analyzed by moisture analyzer. The titratable acidity was determined by mixing 5 mL of the Ethiopian honey wine, *Tej*, sample and 2-mL distilled water, followed by titration with 0.1 M NaOH using three drops of 1% phenolphthalein [13]. The amounts of acid was calculated as follows:

 $$\begin{array}{c} \text{Lactic acid }\left(\text{g}/100\text{mL}\right)=\frac{\text{Amount of NaOH titrated}\times \text{mol}/\text{L of NaOH}\times 5}{\text{Volume of sample }\left(\text{mL}\right)}. \end{array}$$

### 2.4. Isolation and Enumeration Bacteria From Ethiopian Honey Wine, *Tej*

The spread plate method was used for isolation of bacteria from the samples and for estimation of their number. Briefly, 25 mL of each sample was mixed with 225 mL of 0.1% (*w*/*v*) sterile peptone water and serially diluted. Then, 0.1 mL of the final dilution was spread on MRS agar plates. The plates were incubated anaerobically at 37°C for 24–48 h. Colonies were counted as colony-forming units (CFUs), and 10 different colonies per plate were picked from countable MRS agar plates for further analysis. The purity of the colonies was checked by repeated streaking on MRS agar plates. The number of CFUs was calculated as follows:

 $$\begin{array}{c} \text{CFU}/\text{mL}=\text{Number of colonies}\times \text{dilution factor}/\text{volume of inoculum in milliliter}. \end{array}$$

### 2.5. Assessment of Probiotic Properties' LAB Isolates

#### 2.5.1. Determination of Antimicrobial Activity

Antibacterial activity of the LAB isolates against *Escherichia coli* ATTC 25922, *Shigella boydii* ATCC 25931, and *Staphylococcus aureus* ATTC 25913 was determined using agar well diffusion method [14]. Briefly, overnight cultures of these test organisms comparable to the standard turbidity of 0.5 McFarland or 10^8^ cfu/mL were spread on Mueller Hinton agar by sterile swab and four wells (6 mm) were made using sterile cork-borer. Then, 100 *μ*L of 48-h cultured LAB strains' cell-free supernatant obtained by centrifugation (5000 rpm, 10 min, and 4°C) was pipetted into holes or wells in the agar plates containing test strains. The plate was then incubated at 37°C for 24–48 h. Antimicrobial activity was recorded as growth-free inhibition zones around the well.

#### 2.5.2. Bile Tolerance Test

So as to test bile tolerance, overnight LAB cultures (10^8^ CFU/mL) were added to different test tubes containing fresh MRS broth with 0%, 0.3%, 0.5% and 1% bile salt (*w*/*v*), and incubated at 37°C for 6 h. The bacterial suspension was then serially diluted in sterile peptone water, plated on MRS agar, incubated at 37°C for 24 h, and counted. Bile tolerance was expressed as percentage using the following equation: Bile resistance (%) = Number of colonies on the medium with ox gall)/Number of colonies on the medium without ox gall) × 100.

#### 2.5.3. Salt Tolerance Test

Overnight cultures of LAB isolates (10^8^ CFU/mL) were inoculated into MRS broth with varying sodium chloride (NaCl) concentrations (4% and 7%) and were incubated at 37°C for 24 h. The viable cell counts were enumerated by plating onto the MRS agar plates and anaerobically incubated at 37°C for 24 h. Salt-tolerant cells were assessed by calculating the ratio of survived viable cell/cfu on MRS agar plate compared to the control (without salt).

#### 2.5.4. Acid Tolerance Test

Overnight culture of the selected LAB isolates with a final population of 10^8^ CFU/mL were inoculated to 5 mL MRS broth (pH 2 and 3), incubated at 37°C for 2–4 h, and plated on MRS agar plates. The survival rate was calculated as follows:

 $$\begin{array}{c} \text{Survival rate }\left(\%\right)=\frac{N_{1}}{N_{0}}\times 100, \end{array}$$

where *N*_1_ is the viable count of isolates after incubation and *N*_0_ is the initial viable count (0 h).

#### 2.5.5. Cell Surface Hydrophobicity Test

The cell surface hydrophobicity was conducted *in vitro* by measuring the percentage adherence of microbial cells to nonpolar solvent using p-xylene [15]. LAB isolates were incubated anaerobically at 37°C for 24 h, and OD 600 nm was read and adjusted it 1 (A0). A suspension of 3 mL of LAB was mixed by vortexing with 1 mL of p-xylene for 1 min and incubated at 37°C for 1 h. The aqueous phase at the bottom was taken and OD 600 nm was measured (A1). The resulting hydrophobicity was calculated by subtracting A1 from A0, dividing the difference by A0 and multiplying the quotient by 100.

 $$\begin{array}{c} \%H=\frac{\text{A}0-\text{A}1}{\text{A}0}\times 100. \end{array}$$

#### 2.5.6. Antioxidant Capacity Test

The 1,1-diphenyl-2-picrylhydrazyl (DPPH) free radical scavenging capacity of LAB was evaluated according to the method described by Liu et al., [16]. Briefly, 1.0 mL of the sample (CFS) was added to the same volume of an ethanolic DPPH radical solution (0.2 mM). The reaction solutions were mixed vigorously and incubated at room temperature in the dark for 30 min. The control group contained an equal volume of DPPH radical solution instead of the sample. The blank group was uninoculated broth. The absorbance of the solution was measured at 517 nm after centrifugation at 6000 × g for 10 min. The scavenging ability was defined as follows:

 $$\begin{array}{c} \text{Scavenging activity }\left(\%\right)=\frac{\text{control}–\text{sample}}{\text{control}}\times 100. \end{array}$$

#### 2.5.7. Anticholesterol Capacity Test

Overnight culture of LAB isolates at 1% level was inoculated in MRS broth which contained cholesterol (50 *μ*g/mL) and incubated anaerobically at 37°C for 24 h. Then, cells were removed by centrifugation (9000 g for 15 min) and the remaining cholesterol in the spent broth was determined calorimetrically by spectrophotometer at 550 nm [17]. The control included uninoculated MRS broth with cholesterol. The ability of the isolates to remove cholesterol was measured as follows:

 $$\begin{array}{c} \text{Cholesterol removal }\left(\%\right)=\frac{\text{Control}–\text{inoculated MRS with cholesterol}}{\text{Control}}\times 100. \end{array}$$

#### 2.5.8. Antibiotic Susceptibility Tests

Antibiotic resistance was determined by disc diffusion method against six antibiotics, including ampicillin (10 *μ*g), erythromycin (15 *μ*g), gentamicin (10 *μ*g), penicillin (10 *μ*g), streptomycin (10 *μ*g), and tetracycline (30 *μ*g). Then, 100 *μ*L overnight culture of LAB isolates with a final population of 10^8^ cfu/mL was swabbed evenly over the surface of nutrient agar plates and antibiotic discs were placed on it, followed by anaerobic incubation at 37°C for 24–48 h. The zone of inhibition (diameter in mm) around each antibiotic was measured and classified as susceptible (≥ 21 mm), intermediate resistant (16–20 mm) and resistant, *R* (≤ 15 mm) [18].

### 2.6. Identification of Selected Isolates

Eight LAB isolates which showed antimicrobial activity were randomly selected (because of economic shortage) for molecular identification to species level; the other isolates were not identified. LAB cells were cultured anaerobically on MRS media for 24 h at 30°C, and the DNA was extracted from the bacterial colony [10] and the required full length of 16S rRNA gene was amplified by the pair of primer, 27 F: 5⁣′-AGA GTT TGA TCC TGG CTC AG-3⁣′ and 1492 R: 5⁣′GGT TAC CTT GTT ACG ACT T-3⁣′ with the following PCR condition: denaturation at 94°C for 10 min, followed by 32 cycles at 94°C for 30 s, 55°C for 20 s, 72°C for 55 s, and a final extension at 72°C for 5 min in a total reaction volume of 25 *μ*L. The PCR products were sequenced according to previously reported method [10], and the resulting sequences were analyzed and compared against the GenBank database using the basic local alignment search tool (https://blast.ncbi.nlm.nih.gov/Blast.cgi) at the NCBI.

### 2.7. Data Analysis

The experiments were carried out in duplicates; IBM SPSS (Version 25) was used to analyze mean and standard deviation of measurements.

## 3. Results

### 3.1. pH, Titratable Acidity, and Moisture Content of the Ethiopian Honey Wine, *Tej* Samples

The pH values, titratable acidity, and moisture content of the samples varied between 2.94 and 4.66, 0.09 and 0.36 g/l00 mL, and 87.15% and 99.35%, respectively (Table 1).

### 3.2. Isolation, Enumeration, and Identification of LAB Isolated From Ethiopian Honey Wine, *Tej*, Samples

The count of LAB in the samples ranged from 2.5 × 10^7^ (7.4) to 3.5 × 10^6^ cfu/mL (6.5 log cfu/mL) (Table 2). The highest count was obtained from Seka 3 sample and the smallest count was from Chena 2 sample.

### 3.3. Cultural, Morphological, and Biochemical Characteristics

A total of 143 LAB isolates were isolated from 30 *Tej* samples, and characterized by cultural, morphological, and biochemical tests. Based on the cultural and morphological characteristics, all the LAB isolates were smooth in margin and circular in shape and white in color. The elevations were from flat to raised. All isolates were catalase-negative and KOH-negative (Gram-positive).

### 3.4. Molecular Identification and Phylogenetic Tree of LAB

**Figure 1:**
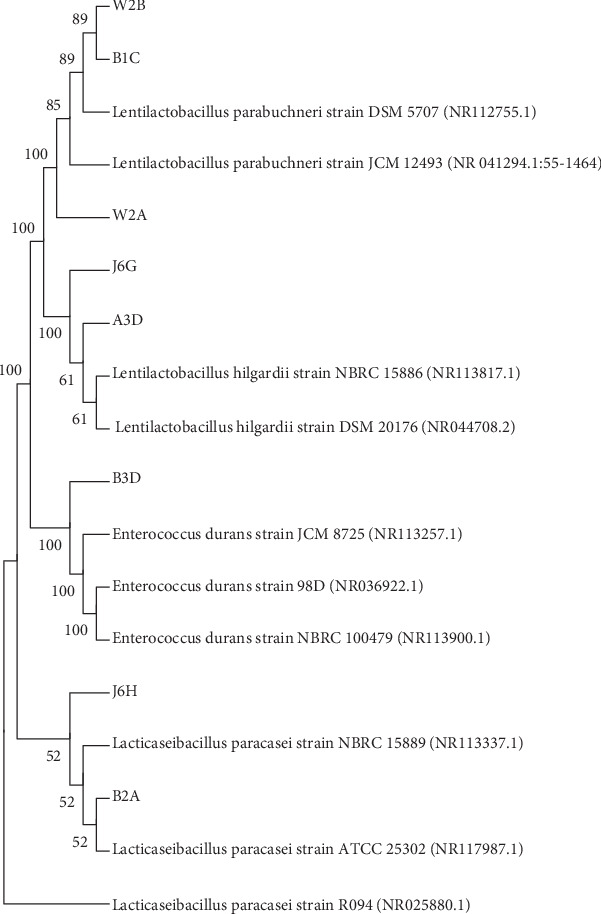
Phylogenetic tree of the LAB isolates.

The species to which the eight identified LAB isolates belong to is indicated in Table 3, and their relatedness to each other and their homologs in the NCBI database is depicted in Figure 1.

### 3.5. Probiotic Properties of the LAB Isolates *In Vitro*

#### 3.5.1. Antimicrobial Activities

From 143 LAB isolates, 37 isolates showed antimicrobial activities (Table 4). They exhibited varying degrees of inhibitory activity ranging from 8 to 18 mm against *E. coli* ATTC 25922 and *S. aureus* ATTC 25913, and from 11 to 19 mm against *Shigella boydii* ATCC 25931. Among all isolates, SK3F showed the greatest inhibition against *Shigella* ATCC 25931 with 19 ± 4.2 mm. The least inhibitory activity was showned by C1 for *E. coli* ATTC 25922 and *S. aureus ATTC* 25913 with 8.0 ± 1.4.

#### 3.5.2. Acid Tolerance

From the 37 tested isolates, seven isolates showed survival rate of ≥ 60% when exposed to pH 2 for 2 h, and three isolates showed survival rate of ≥ 55% when exposed the same pH for 4 h. In pH 3, ≥ 80% each of 28 out of 37 isolates survived after exposure for 2 h; however, J2C, J6C, SK3F, B1C (*Lentilactobacillus parabuchneri)*, C1, M1A, J6G (*Lentilactobacillus hilgardii)*, A1I, and A2H were not able to survive. When the isolates were exposed to pH 3, 35 isolates showed greater than 50% survival rate, and the survival rate of the reaming two isolates was less than 50% (47.2% for A1I and 43.4% for SK3F). The lowest survival rate after exposure to pH 2 for 2 h was observed for J2C (32.6%) and A1I (22%), both which did not survive when exposure time was increased to 4 h.

#### 3.5.3. Bile Tolerance

All the tested 37 isolates showed more than 80% survival rate in 0.3% bile, and 70% in 0.5% bile. In 1% bile, all isolates exhibited more than 50% survival rate, with the highest rate (69%) observed for M2A and J5A (Table 5).

#### 3.5.4. Cholesterol-Lowering Ability

All the 37 isolates showed cholesterol-lowering effect, which ranged from 8% to 54%. The highest cholesterol lowering activity was observed for C2 (54%) and J6C (46%). In addition, J1D, J3B, and SK2C lowered cholesterol level by 42% (Table 6).

#### 3.5.5. Antioxidant Activity

The DPPH free radical scavenging capacity of LAB isolates ranged from 13% for B1D to 33% for SK2B (Table 6).

#### 3.5.6. Hydrophobicity Test

The percentage adherences of microbial cells to p- xylene spanned from 54% by SK2C to 67% by J2A isolates (Table 6).

#### 3.5.7. Antibiotic Susceptibility Test

Antibiotic resistance test against some antibiotics showed various results which were as follows: Isolate M2A was resistance to all antibiotics and isolate J3F was resistant to ampicillin, erythromycin, gentamicin, penicillin, and tetracycline. Isolates SK3F was resistant to erythromycin, streptomycin, and tetracycline. Other six isolates (J1D, J2A, J5G, J6E, J6G (*Lentilactobacillus hilgardii*), and A2B) showed resistance against tetracycline. Intermediate results were recorded against ampicillin by A2H, Sk2C, and C1, against gentamicin by SK2C and SK3F, against penicillin by J1D, J5G, J6G (*Lentilactobacillus hilgardii*), A1I, and A2B, against streptomycin by J1D, J2A, J3F, J5D, J5G, J6E, J6G (*Lentilactobacillus hilgardii*), A1I, A2B, and GMA, against tetracycline by J4B, J5D, J6C, J6H (*Lacticaseibacillus paracasei)*, A1I, A4C, D1D, B1C (*Lentilactobacillus parabuchneri*), GMA, and AM. The rest isolates were susceptible to the tested antibiotics (Table 7).

## 4. Discussion

The pH values showed that all samples had acidic properties. There was a significant difference (*p* < 0.05) in pH values between all the *Tej* samples measured in this study. The differences in titratable acidity values of all Ethiopian honey wine, *Tej*, samples were also significant (*p* < 0.05), and the moisture content of *Tej* samples was between 87.15% and 99.35%. The pH and titratable acidity values of the samples were more or less similar to other studies, with pH between 3.02 and 4.90 and titratable acidity as low as 0.1 g/l00 mL to values as high as 1.03 g/l00 mL. The count of LAB in the samples ranged between 7.4 and 6.5 log cfu/mL. Gas productions from glucose showed that the majority of the isolates were heterofermentative.

LABs have the potential to produce antimicrobial products for inhibiting food pathogens. This antagonistic activity of LAB against food-borne pathogens is a better alternative for health than chemical drugs. According to Schillinger and Lucke [19], > 0.5-mm diameters of clear zone were scored positive for inhibition [19]. In this study, the antimicrobial activities against test pathogens exhibited different degrees of inhibitory activity and the value among all isolates was significant except *Shigella boydii*, ranging from 8 to 19 mm of diameter inhibition. This is more or less similar to another study, in which inhibition zone ranged from 17 to 21 mm [20]. The isolates in this study showed greater inhibition against *S. aureus* compared to other studies [21], in which no isolate showed antimicrobial activity against this organism. The antimicrobial activity of the LAB may be due to the production of acetic and lactic acids, hydrogen peroxide, bacteriocins, and reuterine [22].

The acid tolerance result found in this study is in agreement with previous studies demonstrating that potentially probiotic LABs are able to remain viable when exposed to pH ranges from 2.0 to 4.0 [23]. The surviving potential of our isolates at low pH is somewhat lower than that of other studies, in which the survival rate was 69% and 94% at pH 2 and pH 3 for 3 h, respectively [24], and 98% at pH 2 for Datta isolates [12]. The acid tolerance property enable the LAB to resist and survive the acidic environment of the stomach and to pass into the intestine.

The physiological concentrations of human bile is in the range of 0.3%–0.5% [25]. In the present study, all isolates showed more than 50% survival rate 50% in 1% bile. The result is similar to other studies [20]. LAB survived in bile salts because they may contain BSH, an enzyme that confers resistance to bile [26]. LAB strains which produce a high level of exopolysaccharide also have higher tolerance to bile salts [27].

According to Shah and Liong [28], small reduction in cholesterol level (even 1%) could decrease the risk of coronary heart disease by 2%–3% [28]. In this study, the cholesterol-lowering capacity of some of the tested LAB isolates was around 50%. Cholesterol reduction of the LAB isolates could be due to the ability of the LAB to decrease its solubility, resulting in its reduced uptake from the intestine [29]. It may also be attributed to enzymatic deconjugation of bile acids by BSH. Deconjugated bile acids are less soluble and easily eliminated in the feces from the intestine [8]. Cholesterol can be converted to bile salts to replace the ones lost from the gut through faces, leading to reduced level of cholesterol in the serum [30].

Antioxidant activity reduces the risk of reactive oxygen species increase, leading to the protection of our body against oxidative stress [31]. In this study, the DPPH free radical scavenging capacity of LAB isolates ranged from 13% to 33%. This finding is in consistent with that of a previous study which was conducted on wine and the cell-free extracts with a 12.64%–29.72% scavenging capacity [32].

Hydrophobicity and aggregation are important factors that greatly affect bacterial adhesion. It was reported that LAB strains possessing hydrophobic cell surfaces and aggregation capacity were more capable of adhering to the intestinal cells to perform beneficial effects [33]. In this study, the percentage adherences of microbial cells to p-xylene ranged from 54% to 67%. These figures are lower than the ones reported for LAB isolated from data (99.2%) [12]. According to Sánchez-Ortiz et al., 30% is considered low hydrophobicity (adhesion to p-xylene), 30%–60% is medium, and 60% is high hydrophobicity [34].

In this study, antibiotic resistance were tested against six antibiotics. The majority of the isolates were susceptible to all antibiotics except one isolate. In agreement with other studies [11], most were susceptible to cell wall inhibitor antibiotics (ampicillin and penicillin) and to erythromycin [20]. Antimicrobial resistances of probiotic microorganisms developed by natural or intrinsic resistance, in which case resistance is not transferable; on the other hand, acquired resistance usually caused from bacterial mutation or may carry plasmid encoding of antibiotic resistance genes and potentially transferable to other commensal or pathogenic bacteria [35].

## 5. Conclusion

LABs isolated from different fermented food sources are GRAS and used as probiotic to facilitate certain biological functions of the host. To be used as probiotics they should be selected by safety parameters. The present study described the *in vitro* probiotic properties of LAB isolated from traditional fermented Ethiopian honey wine, *Tej*. In the present study, 37 isolates showed antimicrobial activity and tolerance to various pH (pH 2 and 3) and bile salts (up to 1% conc.) The other probiotic activities which were exhibited by those isolates were great adhesion potential, cholesterol reduction, antioxidant, and antibiotic susceptibility capacity. Five isolates (J2A, SK2B, B2A, and W2B) appear to be the best probiotic potential based on their antimicrobial activity, pH and bile tolerance, and hydrophobicity. So, the potential probiotic isolates which are obtained in this study can be used as probiotics after further identification of the isolates to species level and further study (*in vivo*) is needed.

## Figures and Tables

**Table 1:** The mean value of pH, titratable acidity, and moisture content of Ethiopian honey wine, *Tej*, samples.

**No.**	**Sample**	**pH**	**Titratable acidity**	**Moisture content by %**
1	Jimma/J1	3.5	0.25	96.10
2	Jimma/J2	3.47	0.18	95.80
3	Jimma/J 3	4.66	0.17	96.95
4	Jimma 4/J4	3.42	0.25	94.55
5	Jimma 5/J5	3.89	0.09	85.20
6	Jimma 6/J6	3.7	0.21	98.40
7	Agaroo 1/A1	3.6	0.21	98.30
8	Agaroo 2/A2	3.87	0.15	98.25
9	Agaroo 3/A3	3.85	0.18	96.45
10	Agaroo 4/A4	3.8	0.13	97.70
11	Dedo 1/D1	2.98	0.18	87.70
12	Dedo 2/D2	3.62	0.36	96.60
13	Seka 1/SK1	3.64	0.19	99.25
14	Seka 2/SK2	3.79	0.18	98.35
15	Seka 3/SK3	3.68	0.19	87.15
16	Bonga 1/B1	3.5	0.21	97.30
17	Bonga 2/B2	3.78	0.2	98.85
18	Bonga 3/B3	3.58	0.195	97.65
19	Gimbo/GM	3.71	0.19	99.20
20	Gesha 1/G1	3.66	0.195	98.15
21	Gesha 2/G2	3.63	0.2	98.65
22	Tepi 1/T1	2.94	0.195	97.30
23	Tepi 2/T2	3.06	0.19	98.30
24	Chena 1/C1	3.69	0.2	98.25
25	Chena 2/C2	3.68	0.185	99.25
26	Mizan 1/M1	3.15	0.185	98.60
27	Mizan 2/M2	3.31	0.195	97.95
28	Wushwush 1/W 1	3.7	0.2	95.15
29	Wushwush2/W2	3.56	0.19	98.90
30	Aman/AM	3.01	0.21	99.35

**Table 2:** The mean count of LAB from Ethiopian honey wine, *Tej*, samples.

**No**	**Sample code**	**CFU/mL**	**log cfu/mL**
1	Jimma 1/J1	1.0 × 10^7^	7
2	Jimma 2/J2	2.0 × 10^7^	7.3
3	Jimma 3/J 3	9.6 × 10^6^	7
4	Jimma 4/J4	1.3 × 10^7^	7.1
5	Jimma 5/J5	6.1 × 10^6^	6.8
6	Jimma 6/J6	1.8 × 10^7^	7.3
7	Agaroo 1/A1	6.1 × 10^6^	6.8
8	Agaroo 2/A2	6.0 × 10^6^	6.8
9	Agaroo 3/A3	1.0 × 10^7^	7
10	Agaroo 4/A4	1.7 × 10^7^	7.2
11	Dedo 1/D1	9.9 × 10^6^	7
12	Dedo 2/D2	2.0 × 10^7^	7.3
13	Seka 1/SK1	2.0 × 10^7^	7.3
14	Seka 2/SK2	9.7 × 10^6^	7
15	Seka 3/SK3	2.5 × 10^7^	7.4
16	Bonga 1/B1	6.3 × 10^6^	6.8
17	Bonga 2/B2	9.5 × 10^6^	6.9
18	Bonga 3/B3	7.1 × 10^6^	7
19	Gimbo/GM	1.0 × 10^7^	7
20	Gesha 1/G1	4.2 × 10^6^	6.6
21	Gesha 2/G2	1.1 × 10^7^	7
22	Tepi 1/T1	2.2 × 10^7^	7.3
23	Tepi 2/T2	1.5 × 10^7^	7.2
24	Chena 1/C1	4.7 × 10^6^	6.7
25	Chena 2/C2	3.5 × 10^6^	6.5
26	Mizan 1/M1	5.9 × 10^6^	6.8
27	Mizan 2/M2	5.1 × 10^6^	6.7
28	Wush wush 1/W 1	4.5 × 10^6^	6.7
29	Wush wush 2/W2	9.3 × 10^6^	6.9
30	Aman/AM	4.3 × 10^6^	6.6

**Table 3:** LAB isolates identified to species level.

**Isolate**	**Species identified**
A3D	*Lentilactobacillus hilgardii*
B2A	*Lacticaseibacillus paracasei*
B3D	*Enterococcus durans*
J6G	*Lentilactobacillus hilgardii*
J6H	*Lacticaseibacillus paracasei*
W2A	*Lentilactobacillus parabuchneri*
W2B	*Lentilactobacillus parabuchneri*
B1C	*Lentilactobacillus parabuchneri*

**Table 4:** Mean and standard deviation of antimicrobial activity of LAB isolates.

**LAB species**	**Isolate code**	**Mean and standard deviation of *i*nhibition zone (mm)**		
		***Escherichia coli* ATTC 25922**	***Shigella boydii* ATCC 25931**	***Staphylococcus aureus* ATTC 25913**
Unidentified	J1D	16.0 ± 1.4	13.0 ± 0.00	18.0 ± 1.4
Unidentified	J2A	12.0 ± 5.6	14.0 ± 2.8	12.0 ± 4.2
Unidentified	J2C	13.0 ± 0.00	14.0 ± 1.4	12.0 ± 2.8
Unidentified	J3B	12.0 ± 1.4	12.0 ± 2.8	11.0 ± 2.8
Unidentified	J3F	12.0 ± 0.00	11.0 ± 2.8	13.0 ± 1.4
Unidentified	J4B	18.0 ± 4.2	13.0 ± 0.00	13.0 ± 2.8
Unidentified	J5A	13.0 ± 1.4	14.0 ± 0.00	12.0 ± 0.00
Unidentified	J5D	14.0 ± 4.2	14.0 ± 5.6	14.0 ± 5.6
Unidentified	J5G	14.0 ± 0.00	16.0 ± 1.4	15.0 ± 1.4
Unidentified	J6C	16.0 ± 1.4	14.0 ± 0.00	15.0 ± 2.8
Unidentified	J6E	15.0 ± 2.8	14.0 ± 1.4	14.0 ± 1.4
*Lentilactobacillus hilgardii*	J6G	14.0 ± 1.4	15.0 ± 4.2	14.0 ± 2.8
*Lacticaseibacillus paracasei*	J6H	16.0 ± 1.4	14.0 ± 2.8	17.0 ± 4.2
Unidentified	A1I	13.0 ± 1.4	12.0 ± 0.00	13.0 ± 4.2
Unidentified	A2B	12.0 ± 0.00	13.0 ± 1.4	13.0 ± 0.00
Unidentified	A2H	12.0 ± 1.4	14.0 ± 4.2	13.0 ± 1.4
*Lentilactobacillus hilgardii*	A3D	18.0 ± 0.00	13.0 ± 1.4	17.0 ± 0.00
Unidentified	A4C	16.0 ± 0.00	14.0 ± 2.8	13.0 ± 1.4
Unidentified	D1D	18.0 ± 2.8	18.0 ± 1.4	14.0 ± 5.6
Unidentified	Sk2B	15.0 ± 1.4	16.0 ± 1.4	14.0 ± 2.8
Unidentified	Sk2C	18.0 ± 1.4	13.0 ± 1.4	18.0 ± 0.00
Unidentified	Sk3F	16.0 ± 1.4	19.0 ± 4.2	11.0 ± 1.4
*Lentilactobacillus parabuchneri*	B1C	13.0 ± 0.00	14.0 ± 4.2	12.0 ± 1.4
Unidentified	B1D	13.0 ± 1.4	13.0 ± 0.00	11.0 ± 1.4
*Lacticaseibacillus paracasei*	B2A	18.0 ± 2.8	17.0 ± 2.8	15.0 ± 0.00
*Enterococcus durans*	B3D	12.0 ± 4.2	13.0 ± 4.2	11.0 ± 2.8
Unidentified	GMA	12.0 ± 0.00	14.0 ± 1.4	12.0 ± 2.8
Unidentified	GMB	13.0 ± 4.2	13.0 ± 4.2	12.0 ± 0.00
Unidentified	G1A	10.0 ± 0.00	13.0 ± 2.8	10.0 ± 0.00
Unidentified	T2A	10.0 ± 1.4	12.0 ± 1.4	17.0 ± 2.8
Unidentified	C1	8.0 ± 1.4	14.0 ± 4.2	8.0 ± 1.4
Unidentified	C2	13.0 ± 2.8	14.0 ± 0.00	9.0 ± 1.4
Unidentified	M1A	14.0 ± 1.4	12.0 ± 4.2	11.0 ± 2.8
Unidentified	M2A	9.0 ± 0.00	16.0 ± 1.4	11.0 ± 4.2
*Lentilactobacillus parabuchneri*	W2A	14.0 ± 5.6	13.0 ± 2.8	12.0 ± 0.00
*Lentilactobacillus parabuchneri*	W2B	10.0 ± 1.4	14.0 ± 2.8	9.0 ± 1.4
Unidentified	AM	14.0 ± 4.2	15.0 ± 2.8	13.0 ± 2.8

**Table 5:** Mean of pH value and bile concentration tolerated by LAB isolates.

**LAB species**	**Code**	**pH 2**		**pH 3**		**Bile concentration**		
		**2 h**	**3 h**	**2 h**	**3 h**	**0.3% bile**	**0.5% bile**	**1% bile**
Unidentified	J1D	47.9	26	91.2	82.5	86	77	58
Unidentified	J2A	62.9	51.6	93.5	89.6	88	78	68
Unidentified	J2C	32.6	0	67.6	50.7	86	77	58
Unidentified	J3B	51.5	42.4	84.2	77.1	86	76	59
Unidentified	J3F	52.4	49	85	75.8	84	77	57
Unidentified	J4B	57.7	53.5	92.5	85.1	87	77	67
Unidentified	J5A	52.9	45.5	78.9	67.1	89	79	69
Unidentified	J5D	52.3	39.6	86.2	62.5	85	74	54
Unidentified	J5G	56.4	43.5	79.1	65.9	84	73	52
Unidentified	J6C	43.9	27.4	64.6	50	91	79	68
Unidentified	J6E	56.3	49.2	87	75.2	90	79	68
*Lentilactobacillus hilgardii*	J6G	44.9	42	64.4	52.6	88	78	67
*Lacticaseibacillus paracasei*	J6H	54.2	40.6	74.2	64.2	85	73	54
Unidentified	A1I	22.9	0	56.9	47.2	86	76	55
Unidentified	A2B	60	50.7	83.5	75.2	88	79	67
Unidentified	A2H	54.9	29.4	59.7	50.6	85	76	56
*Lentilactobacillus hilgardii*	A3D	55	44.9	82.7	72.8	86	76	59
Unidentified	A4C	75.3	56.9	86.7	81.9	89	79	67
Unidentified	D1D	55.7	50	87	81.1	88	78	66
Unidentified	Sk2B	58.4	50.6	81	69.6	87	78	67
Unidentified	Sk2C	57.4	42.5	72	52	86	76	60
Unidentified	Sk3F	44.6	18.4	52.1	43.4	87	76	66
*Lentilactobacillus parabuchneri*	B1C	49.1	34.4	63	50	85	75	56
*Leuconostoc*	B1D	53.9	42.8	84.2	72.8	86	75	59
*Lacticaseibacillus paracasei*	B2A	64	59.3	88.8	80.2	90	79	68
*Enterococcus durans*	B3D	54.2	42.3	84.5	73.2	87	78	66
Unidentified	GMA	61.6	51.6	86.6	82.8	86	77	61
Unidentified	GMB	50.8	37.2	85	71.2	87	76	66
Unidentified	G1A	53.9	44.4	81.3	74.4	86	76	56
Unidentified	T2A	58.6	52	88.4	82.6	84	73	52
Unidentified	C1	48.6	40.2	65.8	51.7	84	74	53
Unidentified	C2	56	40.9	74	62.9	83	73	53
Unidentified	M1A	48.3	26.6	56.3	50.7	88	78	66
Unidentified	M2A	63.2	55.8	90	76.2	90	79	69
*Lentilactobacillus parabuchneri*	W2A	57.8	36.8	72.8	50.8	86	75	59
*Lentilactobacillus parabuchneri*	W2B	63.4	50.7	89.3	78.6	87	76	66
Unidentified	AM	56.7	51.3	91.5	77.1	86	76	62

**Table 6:** The mean plus or minus standard deviation of anticholesterol activity, antioxidant activity, and hydrophobicity of some LAB isolates in percentage.

**LAB species**	**Isolate code**	**Cholesterol-lowering activity**	**Antioxidant activity**	**Hydrophobicity**
Unidentified	J1D	42 ± 5.6	24 ± 2.8	55 ± 4.2
Unidentified	J2A	35 ± 1.4	19 ± 1.4	67 ± 1.4
Unidentified	J5A	31 ± 2.8	24 ± 0.00	62 ± 1.4
Unidentified	J6C	46 ± 2.8	21 ± 2.8	61 ± 1.4
*Lentilactobacillus hilgardii*	J6G	31 ± 1.4	16 ± 4.2	56 ± 0.00
*Lacticaseibacillus paracasei*	J6H	38 ± 8.4	27 ± 2.8	58 ± 4.2
Unidentified	A1I	27 ± 1.4	21 ± 5.6	62 ± 1.4
*Lentilactobacillus hilgardii*	A3D	38 ± 1.4	19 ± 1.4	64 ± 5.6
Unidentified	Sk2B	23 ± 4.2	33 ± 4.2	64 ± 2.8
Unidentified	Sk2C	42 ± 7.0	23 ± 1.4	54 ± 4.2
*Lentilactobacillus parabuchneri*	B1C	27 ± 4.2	19 ± 1.4	62 ± 2.8
Unidentified	B1D	8 ± 4.2	13 ± 0.00	60 ± 1.4
*Lacticaseibacillus paracasei*	B2A	23 ± 5.6	18 ± 2.8	62 ± 0.00
Unidentified	GMB	19 ± 2.8	16 ± 1.4	57 ± 2.8
Unidentified	C1	38 ± 4.2	18 ± 0.00	63 ± 0.00
Unidentified	C2	54 ± 4.2	16 ± 4.2	61 ± 4.2
*Lentilactobacillus parabuchneri*	W2A	23 ± 2.8	20 ± 5.6	63 ± 4.2
*Lentilactobacillus parabuchneri*	W2B	23 ± 2.8	20 ± 5.6	63 ± 4.2
Unidentified	AM	27 ± 1.4	21 ± 2.8	64 ± 4.2

**Table 7:** Mean of antibiotic susceptibility test result.

**LAB species**	**Code**	**Ampicillin**	**Erythromycin**	**Gentamycin**	**Penicillin**	**Streptomycin**	**Tetracycline**
Unidentified	J1D	S	S	S	I	I	R
Unidentified	J2A	S	S	S	S	I	R
Unidentified	J2C	S	S	S	S	S	S
Unidentified	J3B	S	S	S	S	S	S
Unidentified	J3F	R	R	R	R	I	R
Unidentified	J4B	S	S	S	S	S	I
Unidentified	J5A	S	S	S	S	S	S
Unidentified	J5D	S	S	S	S	I	I
Unidentified	*J5G*	S	S	S	I	I	R
Unidentified	*J6C*	S	S	S	S	S	I
Unidentified	*J6E*	S	S	S	S	I	R
*Lentilactobacillus hilgardii*	*J6G*	S	S	S	I	I	R
*Lacticaseibacillus paracasei*	*J6H*	S	S	S	S	S	I
Unidentified	*A1I*	S	S	S	I	I	I
Unidentified	*A2B*	S	S	S	I	I	R
Unidentified	*A2H*	I	S	S	S	S	S
*Lentilactobacillus hilgardii*	*A3D*	S	S	S	S	S	S
Unidentified	*A4C*	S	S	S	S	S	I
Unidentified	*D1D*	S	S	S	S	S	I
Unidentified	*Sk2B*	R	S	S	S	S	S
Unidentified	*Sk2C*	I	S	I	S	S	S
Unidentified	*Sk3F*	S	R	I	S	R	R
*Lentilactobacillus parabuchneri*	*B1C*	S	S	S	S	S	I
Unidentified	*B1D*	S	S	S	S	S	S
*Lacticaseibacillus paracasei*	*B2A*	S	S	S	S	S	S
*Enterococcus durans*	*B3D*	S	S	S	S	S	S
Unidentified	*GMA*	S	S	S	S	I	I
Unidentified	*GMB*	S	S	S	S	S	S
Unidentified	*G1A*	S	S	S	S	S	S
Unidentified	*T2A*	S	S	S	S	S	S
Unidentified	*C1*	I	S	S	S	S	S
Unidentified	*C2*	S	S	S	S	S	S
Unidentified	*M1A*	S	S	S	S	S	S
Unidentified	*M2A*	R	R	R	R	R	R
*Lentilactobacillus parabuchneri*	W2A	S	S	S	S	S	S
*Lentilactobacillus parabuchneri*	*W2B*	S	S	S	S	S	S
Unidentified	*AM*	S	S	S	S	S	I

*Note:* susceptible (S) (≥ 21 mm); intermediate (I) (16–20 mm); and resistant (R) (≤ 15 mm).

## Data Availability

The data that supports this study can be obtained from the corresponding author upon reasonable request.
